# Intimate partner violence is independently associated with poor utilization of antenatal care in Arba Minch town, southern Ethiopia: A cross-sectional study

**DOI:** 10.1371/journal.pgph.0002246

**Published:** 2024-01-02

**Authors:** Dagne Deresa Dinagde, Kassahun Fikadu Tesema, Fitsum Wolde, Gudisa Weyessa Heyi, Gizu Tola Feyisa, Agmasie Damtew Walle

**Affiliations:** 1 Department of Midwifery, College of Health Sciences, Mattu University, Mettu, Ethiopia; 2 Department of Midwifery, College of Medicine and Health Sciences, Arba Minch University, Arba Minch, Ethiopia; 3 Department of Midwifery, College of Health Sciences, Dire-Dawa University, Dire-Dawa, Ethiopia; 4 Departments of Health Informatics, College of Health Sciences, Mattu University, Mettu, Ethiopia; University of the Southern Caribbean, TRINIDAD AND TOBAGO

## Abstract

To ensure the best possible health conditions for both mother and fetus throughout pregnancy, skilled healthcare professionals provide antenatal care (ANC) to expectant mothers. Even though the introduction of antenatal care has reduced maternal mortality by 34% since 2002, some atypical behaviors, such as intimate partner violence, have had a significant impact on how often women seek out expert medical treatment during pregnancy. Hence, early identification of such risk factors is very important to decrease maternal mortality from preventable causes. To assess the prevalence and factors of intimate partner violence and associated factors among pregnant women at Arba Minch town, southern Ethiopia. An institution-based cross-sectional study was conducted among 403 mothers who were enrolled from December 1, 2022, to January 30, 2023. The total sample size was allocated proportionately to the number of women attending antenatal care at each public health facility. Thus, systematic sampling was applied. Kobo Toolbox was used for data collection and cleaning, which was then analyzed using IBM SPSS Version 26. Statistical significance was determined at a *p*-value of less than 0.05. In this study area, the prevalence of intimate partner violence among pregnant women was 35% (95% CI: 30.5–39). The associated factors of intimate partner violence were late initiation of antenatal care (AOR = 3.81, 95% CI: 1.7, 6.04), non-autonomous women (AOR = 1.8, 95% CI: 1.18, 3.14), inadequate antenatal utilization (AOR = 3.41, 95% CI: 1.8, 6.2), and a husband with an extra wife (AOR = 6.0, 95% CI: 4.2, 10.57).This study showed that more than one-third of pregnant women in this study area were facing intimate partner violence. Having extra wife, lack of women’s autonomy, less antenatal care utilization and late initiation of antenatal care were associated with Intimate Partner Violence (IPV). Therefore, it is essential to greatly empower women and provide them significant prestige in the home.

## Introduction

### Background

Antenatal care (ANC) aims to support and preserve pregnant women’s optimal health throughout their pregnancies, deliveries, and puerperium so that they can have successful pregnancies and births and raise healthy offspring. Additionally, it offers a chance for nutrition, labor preparation, delivery care, postpartum contraception, and health education on reproductive instruction [1].

Additionally, antenatal care offers services like blood pressure checks, fetal growth monitoring (by fundal height and sonographic evaluation), urine tests (detecting urinary tract infection), iron-folic acid supplements (to prevent anemia), tetanus shots (to avoid tetanus exposure at birth), at least three doses of intermittent preventive treatment with sulphadoxine/pyrimethamine (IPTp) (malaria prevention), deworming after the first trimester (to decrease the risk of anemia), blood group typing if not already done, human immunodeficiency virus (HIV) and syphilis testing, as well as counseling on nutrition, birth preparation, and other topics [2].

In an effort to implement ANC globally, the MMR has decreased by about 44% in the last 25 years, from 385 (359 to 427) in 1990 to an estimated 216 (80% uncertainty band 207 to 249 maternal deaths per 100 000 live deliveries in 2015). From an estimated 532 000 (496 000 to 590 000) maternal fatalities in 1990 to an estimated 303 000 (291 000 to 349 000) in 2015, the annual number of maternal deaths has fallen by 43%. On the other hand, the global lifetime risk of maternal death has decreased significantly, from 1 in 73 to 1 in 180 [3].

However, different previous studies found lots of factors were associated with the utilization of antenatal care (ANC), such as women’s decision-making, perceived quality of care, level of respectful and non-abuse care, partner support, maternal education and occupation, husband education, pregnancy-related bad habits, and intimate partner violence (IPV) [4]. IPV during pregnancy has been identified as a major issue linked to inadequate use of ANC services.

Intimate partner violence (IPV) is defined as abusive or aggressive behavior in a romantic partnership that harms the partners’ sexual, physical, and mental health. It is one of the most common types of gender-based violence. Abuse of any kind by a romantic partner, including physical, sexual, and emotional assault, is considered IPV [5]. Every year, more than 324,000 women have suffered from IPV while pregnant. The WHO report states that 38% of pregnant women worldwide have IPV, with Africa having the highest frequency (33%) [6]. Additionally, developing nations have a greater rate of overall IPV during pregnancy (27.7%) than developed countries (13.3%) do [7]. The prevalence of IPV among pregnant women was 28.74%, 33%, and, Nigeria [8], Kenya [9], and 37% in Ethiopia [10] respectively.

IPV interventions, which are typically carried out through law enforcement or psychotherapy methods, are directed toward both IPV victims and IPV violators. The fifth sustainable development goal (SDG), which calls for gender equality, is important for all the other SDGs calling for the abolition of all forms of violence against women and girls, according to UN Women’s flagship study, "Turning promises into action: Gender equality in the 2030 Agenda" [11, 12].

Studies from other low- and middle-income (LMIC) nations have shown that IPV is linked to poor results for maternal and child health. For instance, different studies showed that pregnancy-related IPV was strongly associated with abortion [13], pre-membrane rupturing and increased risk of underweight baby [14], newborn mortality [15] and due to the incidence of preterm labor, increases the risk of caesarean section and hospitalization during birth [16]. Similar to this, other research revealed that exposure to IPV while pregnant is linked to a higher risk of common mental disorders, postpartum depression, and a poorer quality of life in terms of health [17].

To minimize the effects of violence and to maximize ANC use in Arba Minch town, it is essential to comprehend the relationship between IPV and the use of ANC services, including the role of different components within the ecological model. Since there had never been a study on IPV in the area, it was unknown whether or not it was common during pregnancy in Arba Minch, southern Ethiopia. However, it is suspected that IPV is linked to inadequate use of ANC services. Therefore, the purpose of this study is to investigate how IPV affects the use of ANC services in southern Ethiopia.

## Methods and materials

### Study design and period

**Fig 1:**
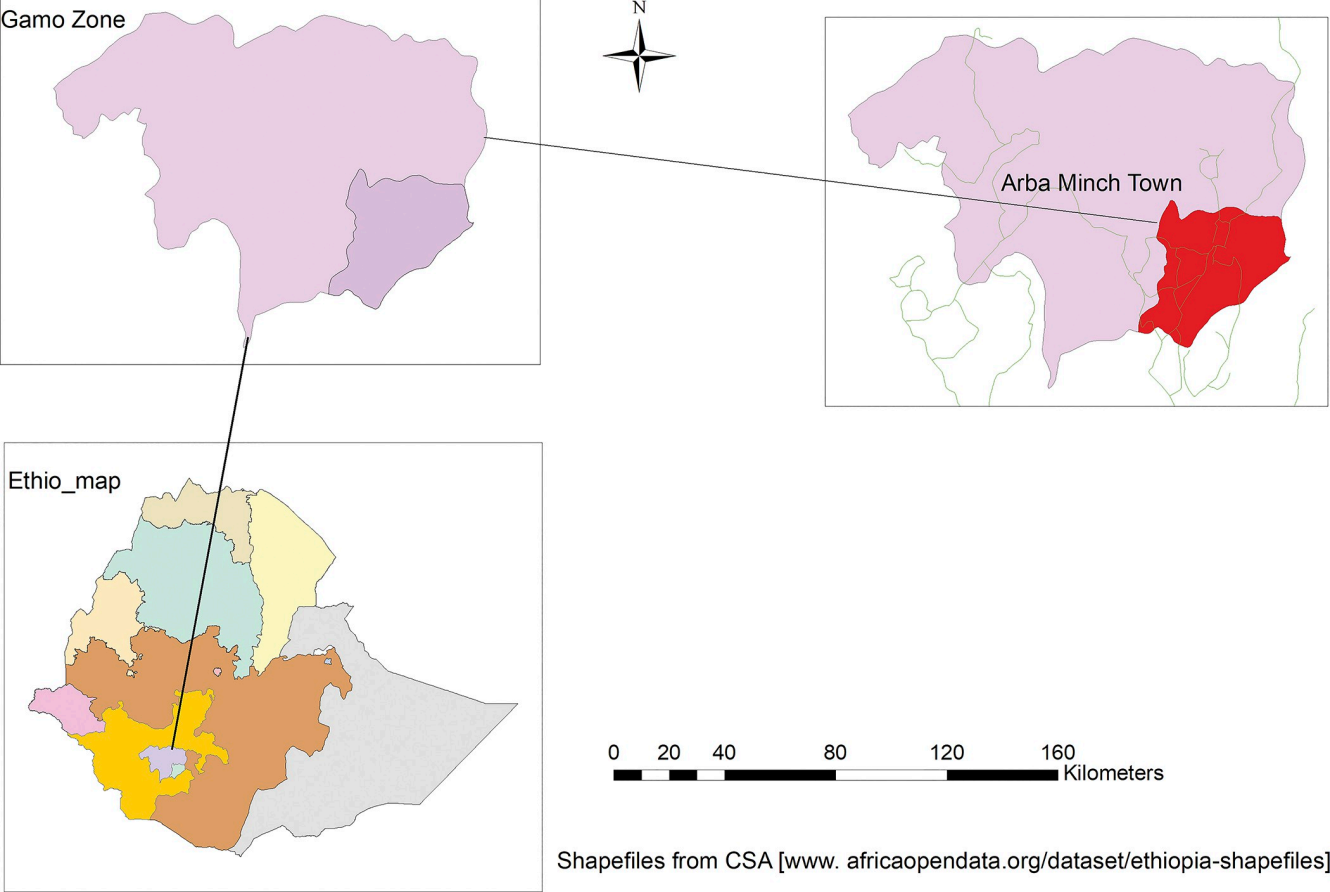
Map of the study area [Shape file source: CSA, 2013; URL: https://www.africaopendata.org/dataset/ethiopia-shapefiles].

Between December 1, 2022, and January 30, 2023, an institutional based cross-sectional study was carried out in Arba Minch town. The town is situated 275 kilometers from Hawassa, the commercial and political hub of the southern area, and 505 kilometers southwest of Addis Ababa, Ethiopia’s capital city. According to the 2020 population projection, Arba Minch town has a total population of 108,956, with 55,568 females and 53,388 males. The town has two public hospitals, one private hospital, and two health centers. All of the health facilities are providing perinatal care. There are 15 nurses and 30 midwives providing antenatal care in those health facilities. The public health institutions in the town are expected to serve more than half a million people in the town and nearby districts [18], see (Fig 1).

#### Study participants

The study participants were pregnant women attending antenatal care in public health facilities of Arba Minch town and were available during the data collection period. Participants willing to provide information were selected through systematic random sampling. Additionally, mothers who were critically ill on the day of data collection and those referred from facilities outside Arba Minch town were excluded from the study.

### Study variables

#### Dependent variable

•Intimate partner violence during the current pregnancy.

#### Independent variables

Socio-demographic variables; age, religion, educational status, residence, monthly income, early marriage, media exposure, decision-making power

Partner behavior:—husband mental problem, husband alcohol use, having extra wife, having child from other women, chewing/smoking

Obstetrics related characteristics of participants:-timely initiation of ANC, adequate ANC, parity and desire of pregnancy.

### Variable measurements

**Intimate partner violence;** If the respondent says “yes” to any one of the ranges of sexually, psychologically, and physically, or any combination of the three coercive acts used against adult and adolescent women, regardless of the legal status of the relationship with current pregnancy, it is considered intimate partner violence [19].

**Women’s decision making power-:** This is one of the key indicators that measure the level of women’s involvement in household decision-making regarding consumption, expenditures, and reproductive choices. It was labeled as having high decision-making power if mother involved in decision-making independently or with others, and it was assigned to have poor decision-making power if never involved in decision-making [20].

**Timely ANC initiation:—**this indicates that the first antenatal interaction occurred before 12 weeks (during the first trimester) [1].

**Adequate ANC utilization:-** Women are regarded to have adequate prenatal care if they did not miss each suggested visit according to new WHO recommendation [21].

### Sample size determination

The sample size was calculated using single population proportion formula: $n={\left(za\right)}^{2}\frac{p(1-p)}{d^{2}}$, considering the following assumptions: prevalence of intimate partner violence was taken from study conducted in Debra Markos town (41.1%) [22] with confidence interval of 95% and, 5% margin of error.


$$n={\left(za\right)}^{2}\frac{p(1-p)}{d^{2}}$$

$$n={\left(1.96\right)}^{2}\frac{0.411(1-0.589)}{0.05^{2}}=372$$

Finally 409 mothers were included in this study by adding 10% non-response rate.

### Sampling technique and procedure

**Fig 2:**
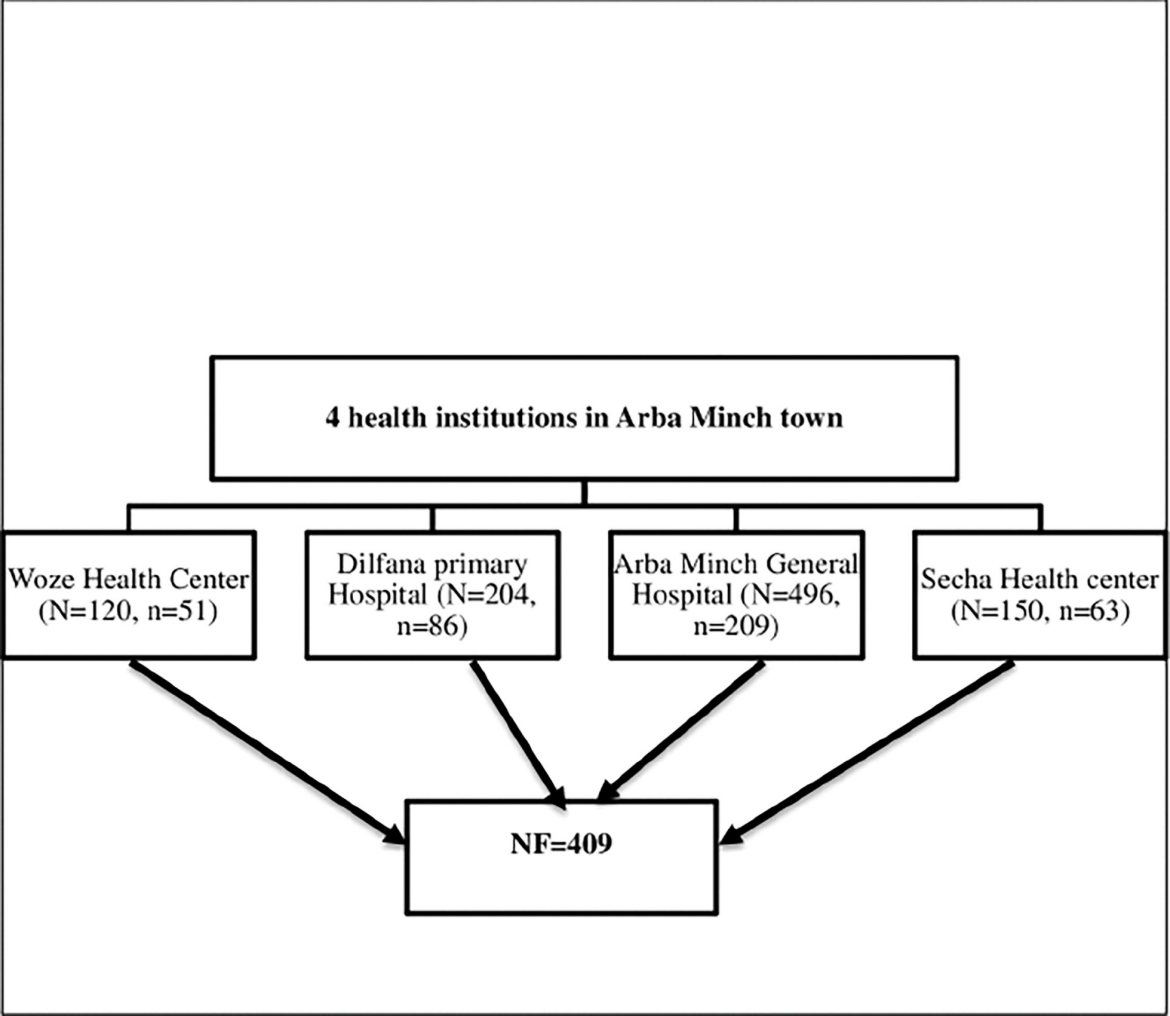
Schematic diagram shows proportional allocation of sample size of each health institution of Arba Minch town.

In Arba Minch town, there are two public hospitals and two public health centers, which provide curative and preventive services for the community. This study considers all public health centers and hospitals in the town. All pregnant women those attending antenatal care in these health care facilities were then enrolled. The sample size for two health facilities was determined according to the proportionate probability technique. Thus, the total participants were identified from the ANC registration book of the previous year for the same months (i.e. December 1 to January 30) from all four institutions. According to the 2021 report of each health facility’s annual ANC report, at similar times in the past two months, 496 pregnant women in Arba Minch general hospital, 204 in Dilfana primary hospital, 150 in Secha health center, and 120 pregnant women attending ANC in Woze health center were used as sampling frame. Then, systematic random sampling was used to reach the respondents. Thus, the first respondent was selected by the lottery method among the 1st kth interval, while the remaining participants of the study were selected by every ‘k’th (i.e. every second) value for all institutions until the total sample size fulfilled (i.e. 409) see (Fig 2).

### Data collection tool and procedure

After being modified from pertinent literatures [4, 23–26] semi-structured questionnaires were used in interviews to collect data, and experts in Arba Minch University’s research committee were involved in the validation of this tool. The tool consists of four sections: socio-demographic, partner behavior, and obstetrics-related characteristics of participants. All pregnant women attending antenatal care in public health facilities in Arba Minch town were enrolled by applying systematic sampling depending on the case flow of the same two months of the previous year, using it as a sampling frame. The questionnaire was designed in English and translated to Amharic language. Four nurses and three midwives were recruited for data collection based on their past experience and fluency in the local language, and they were supervised by two master-holder midwives.

### Data quality management

Following an extensive review of relevant literature and similar studies, a data collection tool was developed to ensure the quality of the data. Properly designed data collection instruments were provided after two days of training for data collectors and supervisors. Pre-testing of the questionnaire was carried out two months before the commencement of the data collection among 20 mothers attending ANC in Shalle health center and all the necessary corrections were made based on the pretest result to avoid any confusion and for better completion of the questions.

### Data analysis and entry

The data was collected by Kobo Toolbox software and imported to the Statistical Package for Social Science (IBM SPSS) Window Version 26 for coding, cleaning, and analysis. Missing values were checked by running frequencies and other data explorations. Descriptive statistics like frequency distributions mean and standard deviation were computed. Bivariate analysis was done primarily to check which independent variables had an association with that of the dependent variable. Independent variables with marginal associations (*p* <0.25) in the bivariate analysis, which are biologically plausible and showed significant association in the previous studies was entered in to a multivariate logistic regression analysis in order to detect association with IPV. The multicollinearity was checked among independent variables and Hosmer-Lemeshow test was used to check the appropriateness of the model for analysis. Finally, adjusted odds ratios (AOR) with 95% CI was estimated to assess the strength of associations and statistical significance was declared at a *p*-value < 0.05 and results was presented using tables, figures, and texts.

#### Ethics statement

Ethical clearance was obtained from the institutional review board of Arba Minch University, College of Medicine and Health Science with reference number of IRB/1328/2022 and the formal letter was sent to all of the town’s medical institutions. Participants were informed of the objectives of the study and its significance before being enrolled. Informed verbal consent was obtained from each participant prior to data collection. The institutional review board approved this approach because some study participants are unable to read or write. Unfortunately, no minor mothers were interviewed for this study; Privacy was guaranteed and no personal information was recorded on any study-related material. Unless otherwise agreed, no one was obligated to take part. Study was conducted according to regulations and guidelines for researches involving human beings.

## Results

### Socio-demographic characteristics of study participants

Out of the 409 study participants, 403 were actually interviewed and provided accurate information, yielding a response rate of 96.2%, while six participants declined to participate in the study as they not will to participate and rushed to leave. The mean age of the women was 29.7 years (SD ± 6) with a minimum and maximum age of 18 and 42, respectively. The majority of the participants, two hundred thirteen (52.9%), were Orthodox Christians. Most women, one hundred sixty (40%), can read and write. The majority of the women, one hundred and ninety four (48.1%), were housewives and one hundred sixty nine (42%) of their husbands can write and read. The majority, two hundred sixty five (66%) of women involved in selection of their husband and Three hundred eight seven (83.6%) of the study participants have a monthly income of more than five thousand Ethiopian birr (Table 1).

**Table 1:** Socio-demographic characteristics of study participants at Arba Minch town (n = 403); 2023.

Variables	Response	Frequency	Percent (%)
Age (in years)	< 20	7	1.7
	20–24	88	21.8
	25–29	100	24.8
	30–34	82	20.3
	35 and above	126	31.3
Residence	Rural	126	31.3
	Urban	277	68.7
Religion	Orthodox	213	52.9
	Muslims	53	13.1
	Protestant	137	34
Maternal education	Unable to read and write	92	22.8
	Able to read and write	160	39.7
	Primary	91	22.6
	Secondary school & above	60	14.9
Maternal occupation	civil servant	80	19.9
	Farming	23	5.7
	house wife	194	48.1
	Traders	106	26.3
Husband education	Unable to read and write	83	20.6
	Able to read and write	169	41.9
	Primary	64	15.9
	Secondary school & above	87	21.6
Who choose husband	She involved	265	65.8
	She doesn’t involved	138	34.2
Income	< 2500 ETB	13	3.1
	2501–5000 ETB	57	13.7
	> 5000	346	83.2

### Partner behavior

Three hundred forty-eight (86%) of the husbands and partners did not smoke, and they also did not chew Kchat. 75.7% of the respondents, three hundred five, stated that they are certain their spouses have no other wives or girlfriends. Three hundred respondents (or 74.4%) stated that their spouses have no children from prior girlfriends or other marriages. Six (6) of the respondent’s husbands have confirmed mental problems. Two hundred fifty-three (62.8%) of the husbands and partners were alcohol users, and the majority, three hundred thirty-one (82.1%) were using TV, radio, or internet (Wi Fi) to get some information.

### Obstetrics related characteristics of participants

Two hundred thirty-four (58.1%) women started to visit antenatal care early within three months of pregnancy, and about one hundred sixty-seven (41.1%) received all recommended contact. The majority, more than two-thirds (74%), wanted or desired pregnancy, sixty-six (16.4%) of women were nulliparous, and more than half (53%) of mothers were autonomous in their health care decisions (Table 2).

**Table 2:** Obstetrics related characteristics among study participants in Arba Minch town, south Ethiopia; 2023 (n = 403).

Variables	Options	Frequency (n)	Percentage (%)
Time of ANC contact	Early	239	59.3
	Late	164	40.7
No of ANC contact	8 and more	167	41.1
	Less 8	236	58.9
Parity	Nulliparous	66	16.4
	Multipara	337	83.6
Wanted pregnancy	Yes	298	73.9
	No	105	26.1
Women’s autonomy	Yes	214	53.1
	No	189	46.9

### Prevalence and form of intimate partner violence during pregnancy

**Fig 3:**
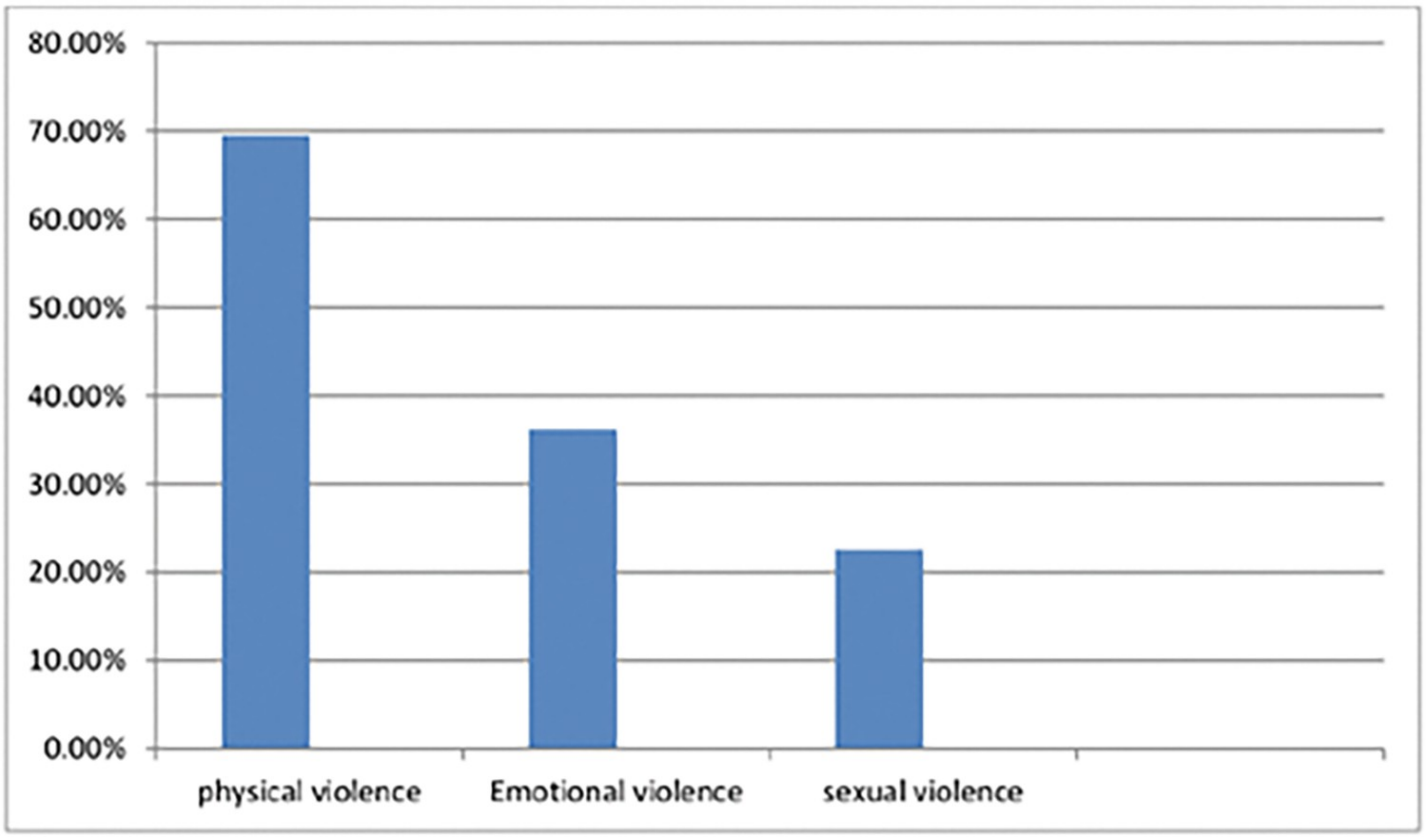
Form of intimate partner violence among women attending ANC at Arba Minch town 2023.

From a total of 403 women booking for ANC, one hundred forty-one (35%) with a 95%CI (30.5, 39), or on the other hand, ninety-eight (69.5%) of the participants claimed physical violence like kicking, slapping, and hitting, fifty-one (36.2%) of the participants claimed emotional (psychological) violence, and ninety-one (22.6%) sexual violence (**Fig 3**).

### Factors associated with intimate partner violence during pregnancy

In the bivariate binary logistic regression, those variables with p<0.25 were candidates for multiple logistic regression, and statistical significance was declared at a p-value < 0.05 as below. Thus, age, maternal education, maternal occupations, income, unwanted pregnancy, exposure to media, mental problems, having extra girlfriends, complete ANC, timely visit ANC, using alcohol, and women’s autonomy were candidates for multivariate analysis.

This study’s multivariable logistic regression analysis revealed that women’s autonomy, timely ANC attendance, and extra girlfriends (other wives) all had a statistically significant association with the outcome variable, IPV.

Those mothers who were not autonomous in economic or health decisions were 80% more likely to face IPV as compared to those who had the power to make decisions related to health, family planning, and economic practice (AOR = 1.8, 95% CI: 1.18–3.14). Those mothers whose husbands were living with a double or extra wife were statistically significant to IPV. These mothers experience IPV six times more likely when compared to those living with one to one husband. Furthermore, those mothers who utilized antenatal care early and adequately were less likely to face IPV (AOR = 3.41, 95% CI: 1.8–6.2) (**Table 3**).

**Table 3:** Multivariate logistic regression analysis result for variables associated with IPV among women attending ANC at Arba Minch health facilities, 2023 (N = 403).

Variables		IPV		COR(95%CI)	AOR(95%CI)	P-value
		Yes (%)	No (%)			
Women’s autonomy	Yes	62(29)	152(71)	1	1	-
	No	79(41.8)	110(58.2)	1.76(1.17,2.70)	1.8(1.18,3.14)	.015*
Maternal occupation	Civil servant	25(31.3)	55(68.7)	1	1	-
	farming	6(26.1)	17(73.9)	0.78(.27,2.21)	0.88(.27,2.84)	.077
	housewife	78(40.2)	116(59.8)	1.47(.85,2.61)	2.13(1.11,4.10)	-
	traders	32(30.2)	74(69.8)	0.95(.51,1.78)	1.24(.61,2.55)	-
Recommended ANC visit	≥8 contact	51(30.5)	116(69.5)	1	1	-
	<8 contact	90(38.1)	146(59.1.)	1.4(.92,2.14)	3.41(1.8,6.2)	.006*
Another wife	No	79(25.9)	226(74.1)	1	1	
	Yes	62(63.3)	36(36.7)	4.93 (3.03,8)	6.0(4.2,10.57)	.000*
Time of ANC	Early	67(28)	172(72)	1	1	-
	Late	74(45.1)	90(54.5)	2.1(1.39,3.2)	3.81(1.7,6.04)	.000*

* = p < 0.05 (statistically significant), 1 = Reference group

## Discussion

According to this study, 35% (95CI: 30.5, 39), of pregnant women experienced intimate partner violence in the study areas. This finding is lower than any others studies conducted in developing countries like Ethiopia, for example study conducted in Iran 2022 which was 51.5% [27], among Afghani in Iran 2021 which was 56.6% [28], sofala, Mozambique 2019 which was (47.3%) [29], Debra Markos, northern Ethiopia 2019 (41.1%) [22]. This may be acceptable. Given that this study focused on metropolitan mothers, where appropriate levels of educated men reside and where crime control is also simpler than in rural areas, again, the possible explanation for the observed variation from a study in Debra Markos town might be due to an intercultural difference between the two settings. Besides, the probable cause of the discrepancy may be the difference in the sample size they used.

However, this finding is much higher than studies conducted in Terai, Nepal, in 2018, which was 28.9% [4], and Bengaluru, India 2021 which was 3.7% [30]. This may be caused by many socioeconomic activities, cultural practices, or even religious practices.

Pregnant women who did not start their ANC within three months of pregnancy were three times more likely to experience intimate partner violence during their pregnancy compared to those who started their ANC early within three months (AOR = 3.18, 95% CI: (1.7, 6.04)).This finding is supported by studies conducted in Rwanda [31], Debra Markos, Ethiopia [22], and North West Ethiopia [32]. This may be because early ANC initiation leads to more visits, which improves the likelihood that women will receive information on sexual and reproductive health, including violence, from healthcare professionals.

The use of adequate antenatal care services was substantially correlated with the absence of intimate partner violence during pregnancy [22] (IPV), as those mothers who do not face IPV during pregnancy are three times more likely to use adequate ANC as compared to their counterparts (AOR = 3.4, 95% CI: 1.3–5.2). This finding is comparable with studies conducted in Timor-Leste, southeast Asia [33], Rwanda [31], Benin [34] and Addis Ababa city, Ethiopia [35]. The might be due to lack of social support from their partners/husbands to inform them on their timely utilization of ANC. Because of this, women who are exposed to utilize less ANC may not modify their behavior for the better to attend ANC appointments on time.

It is essential to women’s independence, privacy, and security that they have the unalienable right to make their own decisions about their sexuality and reproductive health [36]. This study discovered that non-autonomous women (those mothers not involved in decision-making) had an 80% likelihood of experiencing IPV during their current pregnancy (AOR = 1.8, 95% CI: 1.18–3.14). This finding is supported by studies conducted in Rwanda [31] and northwest Ethiopia [37]. This may be because women with control over health care decisions may have greater mobility, fewer financial concerns, and the ability to travel independently. Additionally, there may be a relationship between autonomy and other factors, including women’s education and urban residence, both of which are related to decreasing the likelihood of using IPV.

Even though it has distinct effects on mothers, marriage to many women is still practiced in many cultures and religions. The odds of IPV among women whose husbands have another wife were six times more likely than those among mothers whose husbands have no other wife (AOR = 6.0, 95% CI: 4.2–10). This may be acceptable because their husband is not concerned about the impact of their multiple marriages. This variable was tried to be assessed in different studies but was statistically significant in this study.

### Limitation of the study

The study’s small sample size and cross-sectional design prevented studying causation. Given the subject’s stigmatizing character, there’s a chance that fewer IPV cases may be reported.

## Conclusion

This study showed that more than one-third of pregnant women in this study area were facing intimate partner violence. Having an extra wife, a lack of women’s autonomy, less antenatal care utilization, and late initiation of antenatal care were associated with IPV. Therefore, it is essential to greatly empower women and provide them with significant prestige in the home.

## Supporting information

S1 Data Raw data. (SAV)

S1 File Tools used for data collection. (DOCX)
