# Supplementary material for: Intimate partner violence is independently associated with poor utilization of antenatal care in Arba Minch town, southern Ethiopia: A cross-sectional study
Source: PLOS Glob Public Health. 2024 Jan 2;4(1):e0002246. doi: 10.1371/journal.pgph.0002246 (PMC10760698; doi:10.1371/journal.pgph.0002246)
Supplement: S1 File — (DOCX) [file pgph.0002246.s002.docx]

| NO | **Socio-demographic factors** |  | | | |
| --- | --- | --- | --- | --- | --- |
| 101 | How old are you? | In years ……………….. | | | |
| 102 | Residence | 1. Rural 2. Urban | | | |
| 103 | Monthly income | ---------------ETB | | | |
| 104 | Your religion? | 1. Orthodox 2. Muslim 3. Protestant   Others specify……….. | | | |
| 106 | Your educational level? | 1. Unable to read and write 2. Able to read and write 3. Primary 4. Secondary school & above | | | |
| 107 | Your occupation? | 1. House wife 2. Farming 3. Traders 4. Civil servant   Other specify …………. | | | |
| 108 | Your marital status? | 1. Single 2. Married but Separate 3. Married and live to together 4. Divorced 5. Windowed | | | |
| 109 | If married, Your husband’s educational status? | 1. Unable to read and write 2. Able to read and write 3. Primary 4. Secondary school & above | | | |
| 110 | If married, Your husband’s occupation? | 1. Farming 2. Traders 3. Civil servant   Other specify ………….. | | | |
| 111 | Who choose your husband? | 1. Both, 2. Myself, 3. My family  4. Partner chooses 5.Partner’s 6. Family, 7. my colleague | | | |
|  | **Types of violence** |  | | | |
| 1. | In general, how would you describe your relationship? | 1. a lot of tension  2. Some tension  3. No tension | | | |
| 2. | Do you and your partner work out arguments with: | 1. Great difficulty  2. Some difficulty  3. No difficulty | | | |
|  | Physical violence |  | | | |
| 3. | Do arguments ever result in hitting, kicking or pushing? | 1. Yes 2. No | | | |
| 4. | Has your partner ever abused you physically? | 1. Yes 2. No | | | |
|  | Emotional violence |  | | | |
| 5. | Do you ever feel frightened by what your partner says or does? | 1. Yes 2. No | | | |
| 6 | Do arguments ever result in you feeling down or bad about yourself? | 1. Yes 2. No | | | |
| 7. | Has your partner ever abused you emotionally? | 1. Yes 2. No | | | |
| 8. | Has your partner ever abused you sexually? Or Physically forced you to have sexual intercourse | 1. Yes 2. No | | | |
|  | **Partner’s behavioral characteristics** |  | | | |
| 1 | Is your husband/partner practice bad habit like smoking and chewing? | 1. Yes 2. No | | | |
| 2. | Is your husband alcohol user? |  | | | |
| 3 | If ‘yes’ to question no 2, how often? | 1. Daily, 2. 1x/week, 3. monthly | | | |
| 4 | Is your husband/partner has other girlfriends or lover? | 1. Yes  2. No | | | |
| 5 | Is your husband/partner has child from other girlfriend or lover? | 1. Yes 2. No | | | |
| 6 | Presence of confirmed mental illness? | 1. Yes 2. No | | | |
| 6 | Which type of media you more used? | 1. Radio  2. TV  3. No  4. other------------ | | | |
|  | **Obstetrics related characteristics of participants** |  | | | |
| 1 | At what months of your pregnancy you receive first ANC? | --------------------- | | | |
| 2 | Have you visited all recommended ANC? | 1. Yes 2. No | | | |
| 3 | If ‘No’ to question no 2, why? | 1. Delay registration  2. Discontinued | | | |
| 4 | Parity | 1. Nulliparous 2. multipara | | | |
| 5 | Wanted pregnancy | 1. Yes 2. No | | | |
|  | **Women’s Decision making power** | | | | |
|  | Items | | 3.Respondent herself | 2.Respondent and husband jointly | 1.Husband only |
| 091 | Who usually decides how to spend the income that you bring into the household? | |  |  |  |
| 092 | Who usually decides how to spend the income that your partner brings into the household? | |  |  |  |
| 093 | Who usually decides about making smaller purchases, such as food and other less expensive needs? | |  |  |  |
| 094 | Who usually decides about making more expensive purchases, such as household equipment? | |  |  |  |
| 095 | Who usually decides on which family members you will visit and when? | |  |  |  |
| 095 | Who decides how many children you will have? | |  |  |  |
| 096 | Who usually decides whether your child will be taken for health care to a health facility when s/he is sick? | |  |  |  |
| 097 | Who usually decides whether you or your partner will use any types of contraception, such as condoms or pills? | |  |  |  |
